# Study on the fusion of improved YOLOv8 and depth camera for bunch tomato stem picking point recognition and localization

**DOI:** 10.3389/fpls.2024.1447855

**Published:** 2024-11-29

**Authors:** Guozhu Song, Jian Wang, Rongting Ma, Yan Shi, Yaqi Wang

**Affiliations:** College of Software, Shanxi Agricultural University, Taigu, China

**Keywords:** improved YOLOv8, depth image, fruit stalk recognition, picking point localization, depth value

## Abstract

When harvesting bunch tomatoes, accurately identifying certain fruiting stems proves challenging due to their obstruction by branches and leaves, or their similarity in colour to the branches, main vines, and lateral vines. Additionally, irregularities in the growth pattern of the fruiting pedicels further complicate precise picking point localization, thus impacting harvesting efficiency. Moreover, the fruit stalks being too short or slender poses an obstacle, rendering it impossible for the depth camera to accurately obtain depth information during depth value acquisition. To address these challenges, this paper proposes an enhanced YOLOv8 model integrated with a depth camera for string tomato fruit stalk picking point identification and localization research. Initially, the Fasternet bottleneck in YOLOv8 is replaced with the c2f bottleneck, and the MLCA attention mechanism is added after the backbone network to construct the FastMLCA-YOLOv8 model for fruit stalk recognition. Subsequently, the optimized K-means algorithm, utilizing K-means++ for clustering centre initialization and determining the optimal number of clusters via Silhouette coefficients, is employed to segment the fruit stalk region. Following this, the corrosion operation and Zhang refinement algorithm are used to denoise the segmented fruit stalk region and extract the refined skeletal line, thereby determining the coordinate position of the fruit stalk picking point in the binarized image. Finally, the issue of missing depth values of fruit stalks is addressed by the secondary extraction method to obtain the depth values and 3D coordinate information of the picking points in RGB-D camera coordinates. The experimental results demonstrate that the algorithm accurately identifies and locates the picking points of string tomatoes under complex background conditions, with the identification success rate of the picking points reaching 91.3%. Compared with the YOLOv8 model, the accuracy is improved by 2.8%, and the error of the depth value of the picking points is only ±2.5 mm. This research meets the needs of string tomato picking robots in fruit stalk target detection and provides strong support for the development of string tomato picking technology.

## 1 Introduction

The country takes the lead in terms of the cultivated area of cluster tomatoes, and its production ranks among the highest in the world. Annual production reaches millions of tons, and this figure continues to rise steadily. According to statistics, China’s total cluster tomato production reached approximately 8 million tons by 2023, representing a significant increase from 6.2 million tons in 2022. And it is expected that by 2024, cluster tomato production will surpass the 11 million ton mark. These figures not only reflect the dynamism of the tomato industry but also indicate the growing market demand for tomato products, including smaller varieties of tomatoes (China Report Hall Network, 2024). In the current agricultural context, cluster tomato harvesting is considered a crucial agricultural activity. Fruit and vegetable harvesting is a labor-intensive component of agricultural production that has traditionally relied on manual operations.This approach is not only inefficient but also vulnerable to seasonal variations and climatic conditions. With the advancement of agricultural modernisation, automation technology has become an important means to improve productivity and reduce labour costs. Among them, computer vision technology plays a key role. Traditional computer vision relies heavily on colour and shape recognition, but these methods are susceptible to light and occlusion in complex environments. To address these issues, AI-driven computer vision technologies, especially deep learning, are becoming mainstream. Convolutional neural networks (CNNs) improve recognition accuracy by processing complex image features. In addition, multimodal data fusion techniques help overcome the shortcomings of single vision systems. However, despite the excellent performance of AI techniques in experimental environments, real-world applications still face challenges such as light variations and environmental complexity, and related studies (e.g., Zhang et al., 2020 and Bergerman et al., 2016) have pointed out that further optimisation of recognition techniques is still the key to achieving full automation. In complex natural environments (e.g., light, shadow, shade, etc.), existing techniques may also suffer from recognition errors or fail to accurately determine the picking point. Moreover, automated equipment needs to consider diverse scenarios and conditions in actual operation, and current technology may be difficult to meet the demand for accurate picking in all situations. Therefore, there is an urgent need to further develop and improve related technologies to achieve accurate identification and guidance of tomato picking points under different conditions. Advanced artificial intelligence and computer vision technologies are used to automatically identify and determine the picking location of ripe bunches of tomato stalks. This approach not only helps to reduce labour pressure and improve productivity for farmers, but also significantly reduces production costs and improves the quality and yield of produce. Simultaneously, picking point identification and positioning technology has a broad range of application areas. It is not only applicable to the picking of crops such as fruits and vegetables but also provides accurate picking guidance for automated equipment. This technology reduces losses in the picking process and improves the utilization of agricultural products, thereby promoting the development of the agricultural industry in the direction of intelligence and efficiency.

The identification and localization of picking points for ripe cluster tomatoes depend on the predictive localization of fruit shape features and the localization of fruit stalk picking points based on the relationship between stalk and fruit position. In a related study, Montoya Cavero proposed a deep learning pepper recognition and pose estimation framework. The framework utilizes high-resolution colour images from an RGB-D based active sensor to detect and segment individual green, red, orange and yellow peppers and their pedicels (which produce stems) on a pixel-by-pixel basis using a mask and a region-based convolutional neural network. Subsequently, the 3D position of the peppers and the z-axis orientation of the camera’s reference system are estimated using depth information from the sensor. The detection accuracy was 60.2% and the position estimation errors obtained by the vision subsystem were x: ±28.75 mm, y: ±21.25 mm, ±15 mm, and the z-axis orientation of the camera’s reference system was ±9.6° (Cavero and Enrique, 2021). Jin Y developed an accurate picking point localization method for horizontally terraced grapes, using a combination of far-view and near-view depth data features. Utilizing depth point cloud data, key points—far-view, near-view, and picking points—were identified based on grape cluster characteristics and the terraced environment. In field experiments focused on near-view localization, the algorithm averaged 0.29 seconds per run, with only 5 out of 100 samples failing in accurate localization (Jin et al., 2022). Kounalakis N employed deep learning to identify ripe tomatoes and their stalks, using depth information to guide the robotic arm in picking point identification. Real-world experiments showed a 65% success rate in recognition and 92.6% accuracy in vision processing for picking point localization (Kounalakis et al., 2021). Sun T proposed a method employing deep learning and active perception for robots in environments with occlusions and varying lighting, achieving a 90% success rate in picking and a 16% average occlusion estimation error after 300 trials (Sun et al., 2023). Paul A utilized YOLO algorithms for pepper detection and a RealSense D455 camera to determine picking point coordinates (Paul et al., 2024). Benavides M explored the YOLOv8s model, achieving a mAP of 0.614 for pepper detection, and developed a CVS for automating stalk recognition in greenhouse tomatoes (Benavides et al., 2020). Suguru Uramoto et al. processed colour images captured by a depth camera with the aim of detecting red ripe large tomatoes and oval mini tomatoes. They then used the depth data captured by the depth camera to calculate the 3D coordinates of the centre of the fruit and the diameter of the fruit. Experiments with tomatoes grown in facility horticulture showed a 96.3 per cent accuracy in their identification (Uramoto et al., 2021). Shuai, L developed a method for detecting tea shoots and keypoints as well as picking point localisation in complex environments. Tea leaves were recognised using the YOLO-Tea model, which improved the mean accuracy (mAP) value of tea shoots and their keypoints by 5.26% compared to YOLOv5. In the inference phase of the model, an image processing method is used to locate the location of the picking point based on the key point information (Shuai et al., 2023). Xiong, J constructed a vision system for lychee image acquisition and proposed a nighttime lychee recognition method and a picking point calculation method. This analysis was first combined with a one-dimensional random signal histogram using an improved fuzzy clustering method (FCM) to remove the background of the nighttime image instead of the lychee fruits and stems. The Otsu algorithm was then used to segment the fruit from the stem base. Harris corners were used for picking point detection. The rate of change in horizontal and vertical position between corner points is analysed to identify picking points. Experiments show that the accuracy of nighttime lychee recognition is 93.75% and the average recognition time is 0.516 s. The highest accuracy of picking point calculation is 97.5% and the lowest is 87.5% at different depth distances (Xiong et al., 2018). In order to address the challenge posed by the similarity in colour between string tomato fruit stalks and their main vines, lateral vines, and branches, coupled with the irregular orientation of the fruit stalks, making it difficult to precisely delineate the fruit stalk area; and considering the limitations of depth cameras in capturing the depth of slender or shorter fruit stalks, leading to significant errors or complete loss of data, a method for identifying and localizing string tomato picking points, based on FastMLCA-YOLOv8 and RGB-D information fusion, is proposed. This method is aimed at preventing the end-effector from erroneously cutting the main or side vines while harvesting bunch tomatoes (Suresh Kumar and Mohan, 2023).

This study focuses on two aspects of identification and localization of bunch tomato fruit stalk picking points.(1) The YOLOv8 model was improved to generate the FastMLCA-YOLOv8 target detection model, facilitating rapid identification of fruit stalks by leveraging the connectivity between bunch tomatoes and their respective stalks.(2) The improved K-means algorithm (using K-means++ to determine the initial clustering centre and Silhouette coefficients to find the optimal number of clusters), morphological corrosion operation and skeletonization zhang refinement algorithm are used to segment, denoise and refine the skeletal lines of the fruit stalk image, so as to further determine the coordinate position information of the picking point on the colour image. Additionally, to address the issue of missing depth values resulting from excessively thin or short fruit stalks, a secondary extraction method is employed to obtain and correct the depth values, thereby acquiring comprehensive location information for the picking points.

## 2 Materials and methods

### 2.1 Data collection and labelling

#### 2.1.1 Data collection

**Figure 1:**
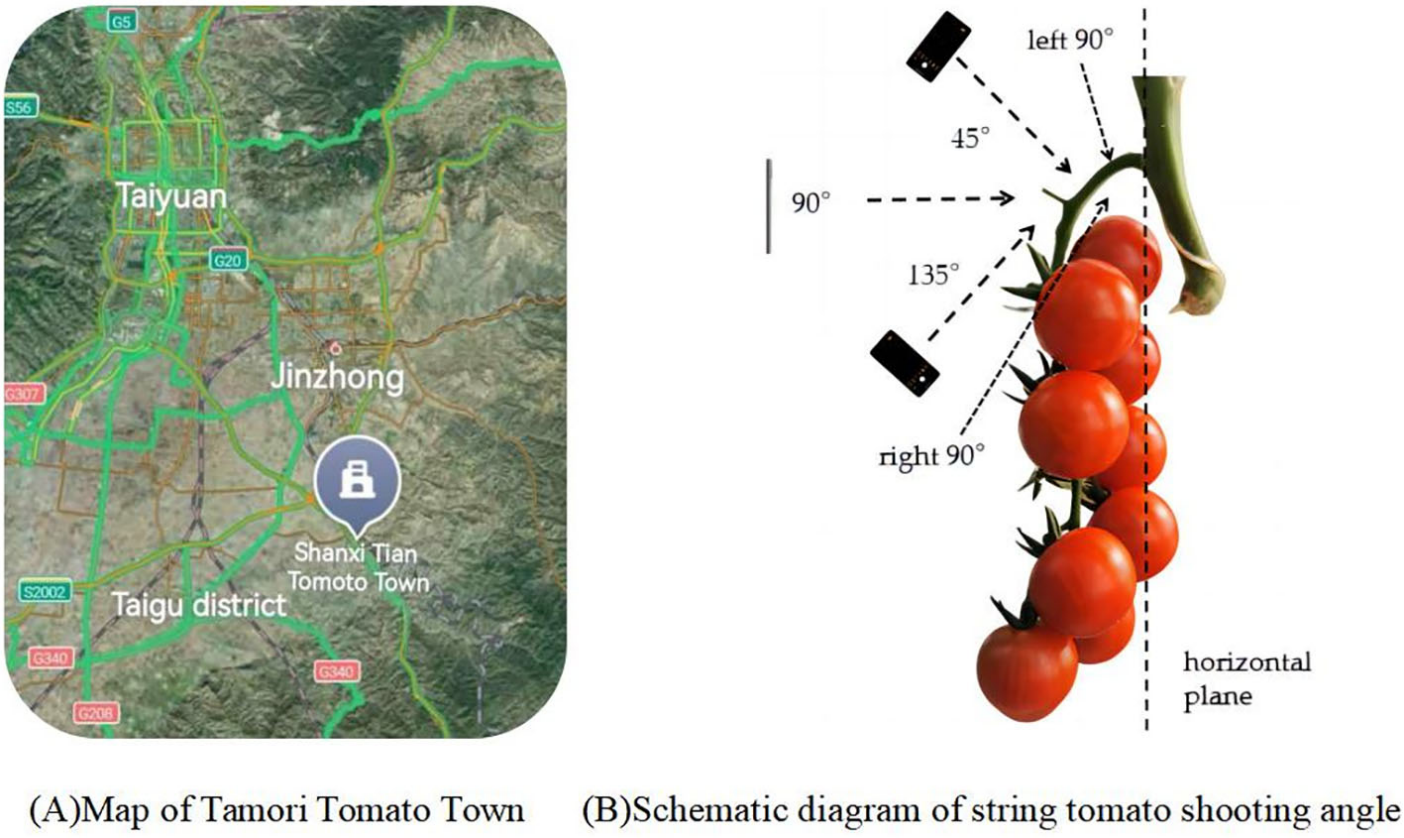
Schematic diagram of string tomato shooting angle.

The data samples for this study were collected from Gertou Village, Fancun Town, Taigu District, Jinzhong City, Shanxi Province (Latitude: 37.4110°N, Longitude: 112.5625°E), where the Melia tomato variety is cultivated, the map is shown in **Figure 1A**. The greenhouse, serving as a hub for integrating and demonstrating advanced tomato production technologies, is recognized as the largest independent continuous glass greenhouse in Asia. Due to the close proximity and high density of the shooting environment, specific requirements were mandated for camera resolution and focal length. Consequently, iPhone 13 Pro Max, vivo X60, and Huawei P40pro smartphones were selected for this study. The cameras, boasting a 2778×1284 12-megapixel resolution and a 77 mm telephoto lens, are adept at swiftly capturing high-resolution images of fruiting peduncles in intricate settings (Montoya-Cavero et al., 2022), as well as obtaining high-quality, undistorted data from various perspectives and angles to satisfy the research requirements.

**Figure 2:**
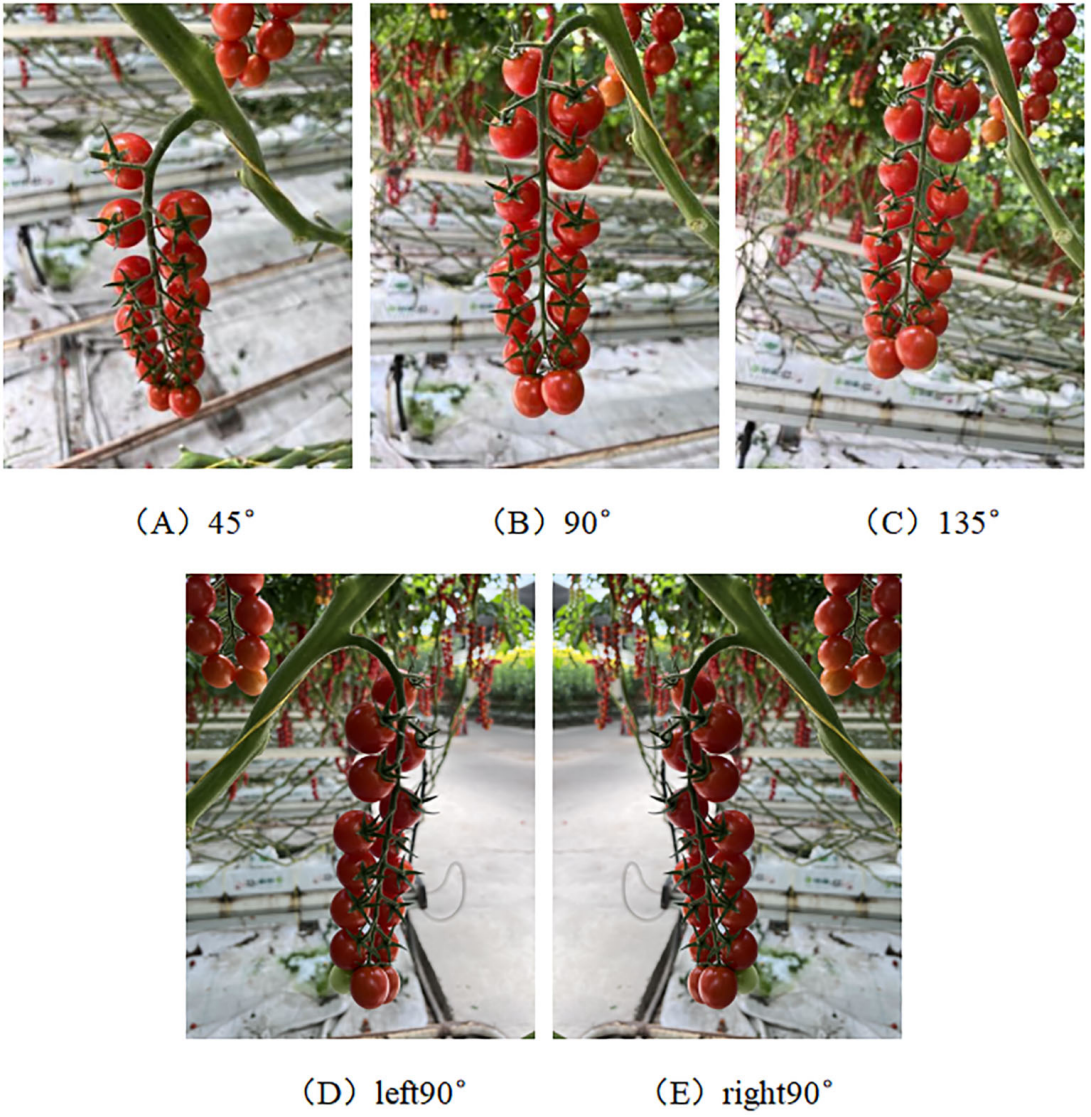
Sample diagram of a fruit stalk shot. Panels **(A–E)** represent the shooting angles 45°, 90°, 135°, left 90° and right 90°.

Data were collected from 15 July 2022 to 31 July 2022 and 28 February 2024, when most of the bunch tomatoes in the greenhouse were ripe and ready for picking. To ensure the diversity of bunch tomato peduncle data, peduncles were photographed under different weather conditions (sunny or cloudy), lighting conditions (front or backlight), time periods, and angles. For different angle conditions, a plane parallel to the fruit stalks and perpendicular to the ground was selected as the reference plane, and the fruit stalks were photographed from three different angles, namely 45°, 90° and 135°. The shooting angle diagram is shown in **Figure 1B**. At the same time, in order to ensure that each fruit stalk can be captured in a complete image, let the camera is facing the fruit stalk to the left and to the right 90° direction to take a photo each, so that each fruit stalk data 5 complete photos, as shown in **Figure 2**. Make sure that a new fruit stalk is observed in each image. Compare each image with the previously obtained image, if they agree then the fruit stalk is complete, otherwise it means that the fruit stalk is damaged or impaired. The collected images were uniformly stored in JPG format and the size was set at 3000 pixels by 4000 pixels. The size of the dataset was 10180,under different conditions,5320 photographs under sunny days, 4860 photographs under cloudy days, 7640 photographs under smooth light, 2540 photographs under backlight, and 2036 photographs under each angle.

#### 2.1.2 Data labelling

**Figure 3:**
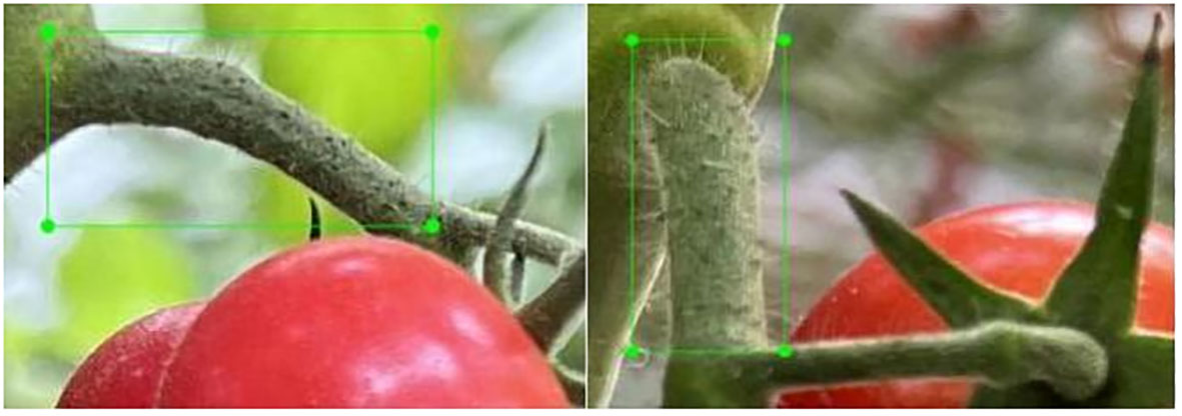
Demonstration of fruit stem labelling.

Following the collection of sample data, images of fruit stalks were systematically sorted, labelled, and utilized to construct a dataset for fruit stalk detection. The open-source LabelImg data annotation tool was employed to label the fruit peduncle data within a bounding box, designating the peduncle as ‘stem’ in accordance with standard annotation standards, with the detailed annotation schematic provided in **Figure 3**. The annotation file containing comprehensive information on fruit peduncles is automatically generated based on the annotation results once the annotation is completed. During the annotation process, particular attention was given to ensure that excessively short or obstructed fruit stalks were not annotated. If a branch resembled a fruit stalk, it was labeled as such. Images failing to meet the annotation standards were excluded to ensure the dataset’s accuracy.

### 2.2 Experimental environment

The configuration parameters of the experimental equipment utilized in this study are delineated in **Table 1**. The processor is a 13th Gen Intel(R) Core(TM) i7-13700K×24. The graphics card is an NVIDIA GeForce RTX 3090. The graphics card’s driver version is NVIDIA-SMI 535.161.07. The system memory is 64GB DDR5. The operating system employed is Ubuntu 22.04.3 LTS. The depth camera utilized is an Intel RealSense D455. The depth image boasts a resolution of 1280x720 and a maximum frame rate of 90 frames per second. The development language employed is Python 3.9.7. The configuration environment’s CUDA version is CUDA 11.5.r.5. The Anaconda version is 4.10.3.

**Table 1:** Configuration parameters of the experimental environment.

Configuration name	Parameters
Processing Unit	13th Gen Intel(R) Core(TM) 17-13700K×24
Display Card (computer)	NVIDIA GeForce RTX 3090
Graphics Card Driver	NVIDIA-SMI 535.161.07
Random Access Memory RAM	64G
Development Language	Python 3.9.7
Deep Learning Frameworks	Tenseflow
Image Acquisition Equipment	iPhone 13 Pro Max, vivox60, Huawei P40pro
Depth Camera(Getting depth values)	Intel RealSense D455
CUDA	CUDA 11.5.r.5
Anaconda	Conda 4.10.3

### 2.3 Experimental process

**Figure 4:**
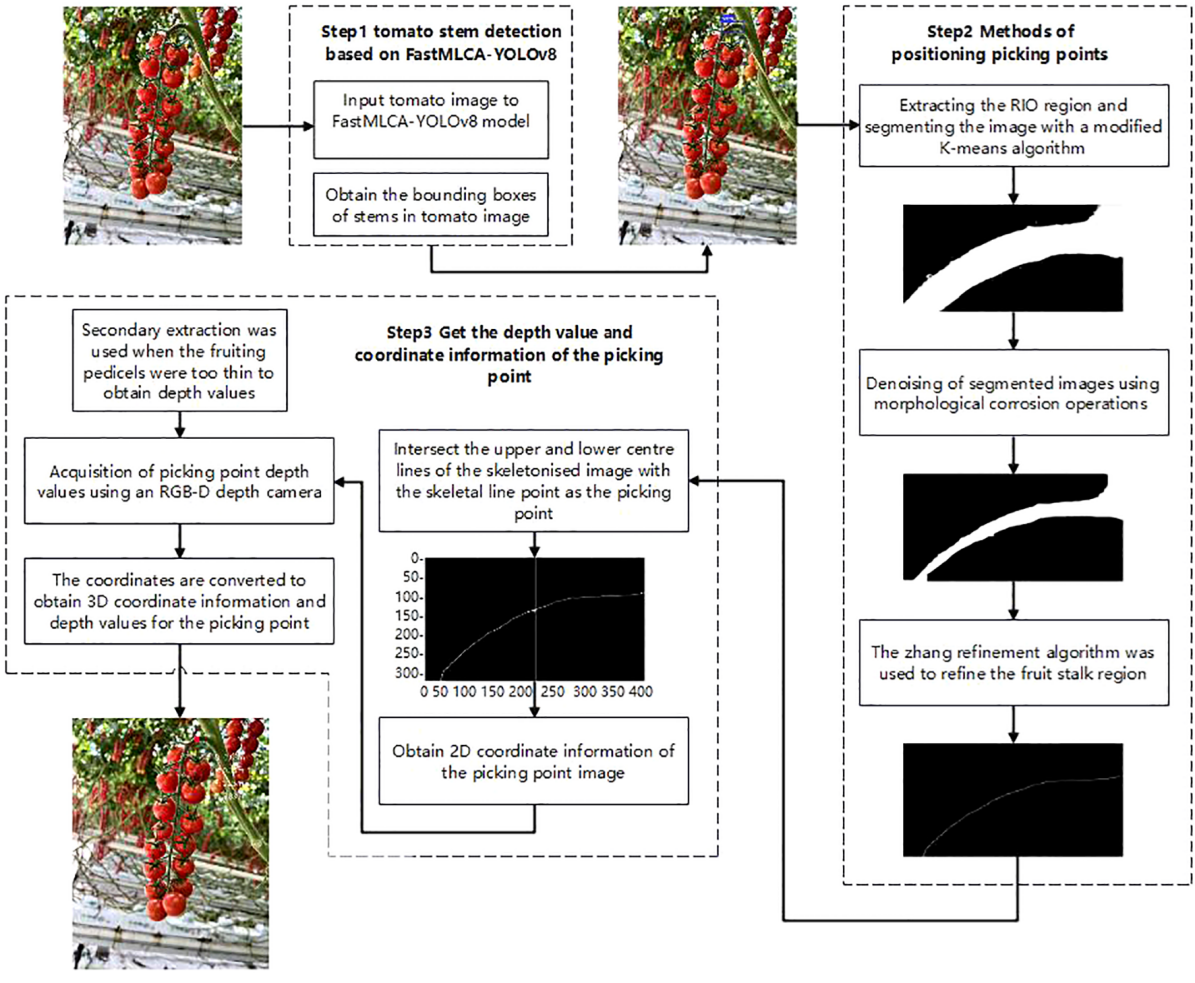
String tomato picking point identification and positioning process.

To address the challenge of identifying and localizing picking points for bunch tomatoes in greenhouse environments, this study introduces an innovative method. This method utilizes the FastMLCA-YOLOv8 target detection algorithm and RGB-D information fusion technology (Arad et al., 2020; Fu et al., 2020) to accurately identify and localize picking points for bunch tomatoes (Klaoudatos et al., 2019), enhancing both the stability and accuracy of the identification process and reducing the risk of the end-effector mistakenly severing the main or lateral vines during the cutting and clamping of bunch tomatoes, thereby improving both the efficiency and quality of the automated picking process (Rong et al., 2022, 2023). The FastMLCA-YOLOv8 target detection model rapidly identifies the minimal rectangular area enclosing harvestable fruit stalks. Subsequently, the viable fruit stalk region of string tomatoes is extracted. The K-means algorithm is then improved by using the K-means++ algorithm to determine the initialized cluster centers. Subsequently, the sum of the squares of the shortest distances from all the remaining samples to the existing cluster centers is calculated, and the next cluster center is selected based on this probability distribution (Solak and Altinişik, 2018). Cyclically try different numbers of clusters k, calculate the Silhouette scores at each value of k, and select the number of clusters with the maximum average Silhouette score as the optimal number of clusters (Shi et al., 2021). Finally, the K-means object is reinitialized and fitted to the data to obtain the labels and then revert them to the shape of the image. This is followed by de-noising and refinement of skeletal lines performed on the segmented image of the improved K-means algorithm using morphological corrosion operations and skeletonisation zhang refinement algorithm, so as to further extract the skeletal lines of the fruit peduncle and to determine the information about the position of the coordinates of the picking point on the colour image. The RGB-D depth camera then determines the picking point’s depth value, with the complete coordinate information obtained following transformation and correction. The algorithmic steps are illustrated in **Figure 4**.

#### 2.3.1 YOLOv8 model

The YOLOv8 model is a more advanced SOTA model that builds on the success of previous YOLO versions and incorporates new features and optimizations designed to further enhance its performance and adaptability (Jocher et al., 2023). Specific innovations include the introduction of a new core network YOLO-NAS (Neural Architecture Search), an innovative anchor-free detection header, and a new loss function YOLOv8 Loss.These enhancements enable YOLOv8 to operate efficiently across various hardware environments, including both CPU and GPU, while achieving substantial improvements in target detection accuracy and speed. The SOTA model consists of a target detection network with resolutions of P5 640 and P6 1280, and an instance based on the YOLACT technology segmentation model; with the same models as YOLOv5, these include N/S/M/L/X models to suit different scene requirements (Li et al., 2024).

The YOLOv8 algorithm is a fast object recognition method that consists of input, Backbone, Neck and output segments: the input section is mainly responsible for the processing of mosaic data, adaptive computation of anchors, and adaptive filling of grey scales of the input image. The core architecture of the YOLOv8 network is composed of Backbone and Neck modules together. The input image is co-processed by several Conv and C2f modules for the purpose of extracting feature maps at various scales. The C2f module is actually an optimisation of the original C3 module, which is the module mainly used for residual learning. It incorporates the advantages of the ELAN structure of YOLOv7 by reducing a standard convolutional layer (Li et al., 2024). The Neck layer is designed based on the FPN+PAN architecture, which is done to improve the performance of the model in terms of feature fusion. The structure contains a local region within each layer and establishes connectivity between each layer. This structure allows for the successful merging of the upper and lower feature maps through upsampling and downsampling, and speeds up the transformation between semantic and localised features. Using this technique, the network has the ability to more efficiently integrate the features of objects at various scales, which in turn enhances the detection of objects at various scales. The detection head of YOLOv8 adopts the common practice of separating the classification head from the detection head. It covers loss estimation as well as filtering functions for the target detection frame. For loss estimation, the TaskAlignedAssigner method is used to determine the distribution of positive and negative samples. Positive samples are selected based on a weighted combination of classification and regression scores. The calculation of loss is divided into two main parts: classification and regression, without involving the objectivity branch. In addition, the YOLOv8 model employs a mosaic-free enhancement strategy in the last 10 epochs of the training phase. This practice aims to reduce the interference of data enhancement on model training so that the model can focus more on processing real test data, thus improving the final detection accuracy and performance.

YOLOv8 uses a task alignment distributor to compute a task alignment metric from classification scores and regression coordinates. The task alignment metric combines the values of classification score and joint intersection (IoU), aiming to achieve simultaneous optimisation of classification and localisation while suppressing low-quality prediction frames (Chen et al., 2024). In the field of object detection, the joint intersection (IoU) is a widely adopted metric that is used to distinguish between positive and negative samples and to evaluate the relative distance of the prediction frames from the ground reality. When the value of IoU exceeds 0.5, the object is usually classified as having been detected. The specific formula is shown in Equation 1.


(1) $$IoU=\frac{\left|A\cap B\right|}{\left|A\cup B\right|}$$

Where A represents the area of the predicted frame and B represents the area of the actual frame. A∩B represents the intersection area of A and B. A∪B represents the area that is the union of A and B.

The algorithm for YOLOv8 comprises inference and subsequent processing steps:

1. Converting the integral form from bbox to bbox 4d; converting the bbox branch generated by Head and using operations of softmax and conv to convert the integral pattern to bbox 4d format;
2. Dimensionality change: YOLOv8 outputs feature maps in three different scales: 80x80, 40x40 and 20x20. in Head, feature maps are presented in six different scales for classification and regression;
3. Decoding recovers the size of the original image: the branches of the classification predictions are computed using sigmoid, and the branches of the prediction frames have to go through a decoding process in order to recover the actual original image in decoded xyxy format;
4. Filtering operation for thresholding. Each image is traversed in batch and threshold filtering is performed using the score_thr method. In this process, multi_label and nms_pre are also considered to ensure that the number of filtered detection frames does not exceed nms_pre;
5. Restore to the original image size and nms: on the basis of the pre-processing process, the remaining detection frames can be restored to the original graph scale before the network output with nms. The number of detection frames generated at the end must not exceed max_per_img.

#### 2.3.2 Improvements to the YOLOv8s model

In this paper, prior to choosing to improve the YOLOv8 model (Wang, et al., 2023), the YOLOv8 model was compared with other YOLO models under the same rounds of training on the same dataset. The results (e.g., **Table 3**) indicate that the YOLOv8 model has the following significant advantages over other models and is more suitable for this study. Firstly, it performs well in handling targets with different scales and complex backgrounds, which is compatible with the complexity of the string tomato picking scene. Secondly, its pre-training accuracy is higher than that of other models. Thirdly, the GFLOPS of the YOLOv8 model is smaller than that of other models, and it has a fast recognition speed and good performance. Therefore, this study improves the YOLOv8 model and proposes a FastMLCA-YOLOv8 based feature extraction and classification model. The bottleneck component of the c2f module has been substituted with that of Fasternet, resulting in the c2f-faster module. Fasternet's bottleneck architecture provides superior parameter optimization capabilities, which improve not only network performance and detection precision but also increase the model's training and inference speed, thereby enhancing the system's real-time functionality (Guo et al., 2024). Concurrently, this bottleneck architecture enables more effective integration of disparate layer feature information, thereby providing a more nuanced and precise feature representation capability that improves target detection accuracy.

**Figure 5:**
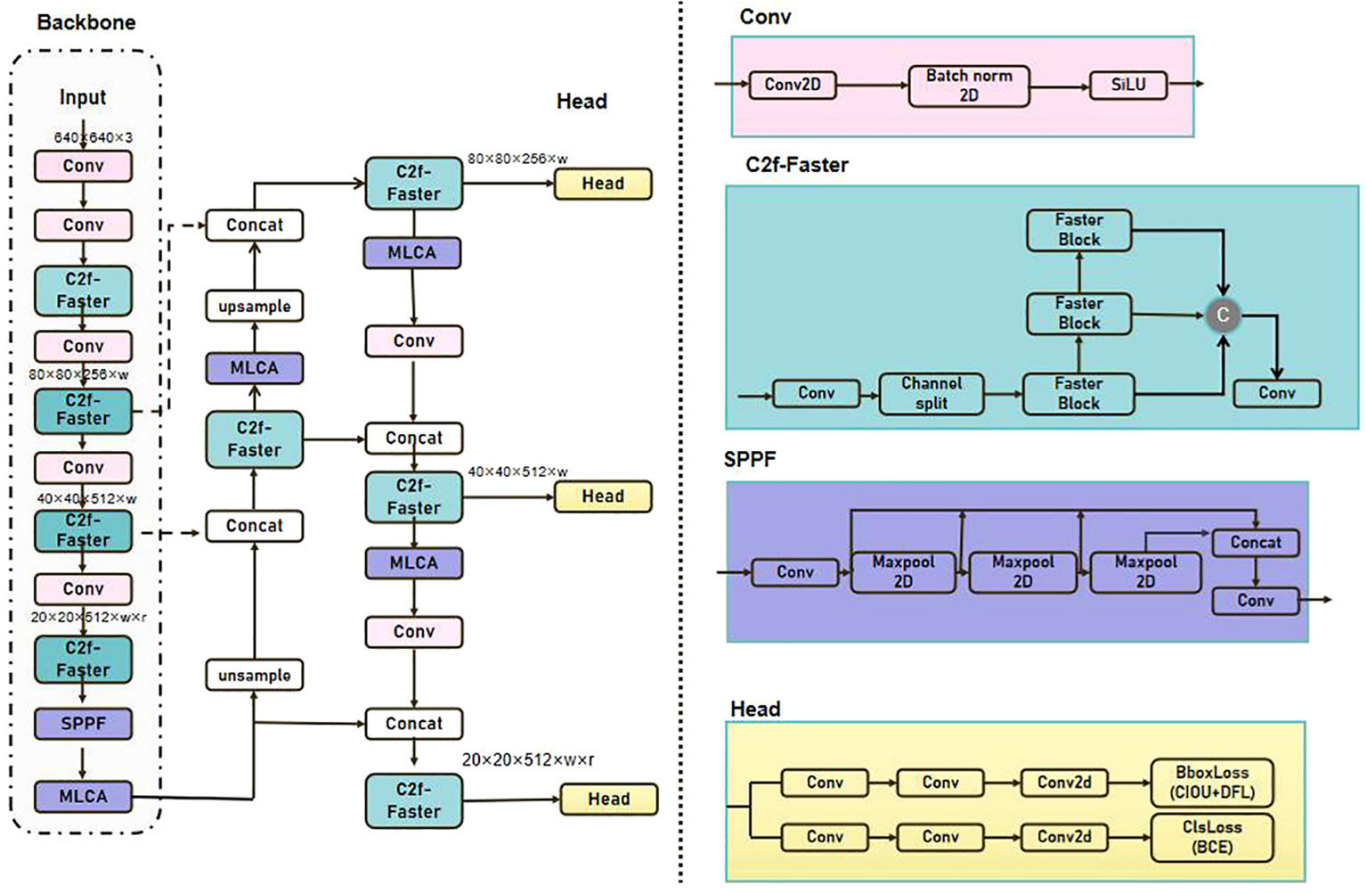
Structure of FastMLCA-YOLOv8.

Based on the c2f module, the MLCA attention mechanism is then added after the backbone network as a way to improve the model’s attention and accuracy to the target. The MLCA attention mechanism enables the model to concentrate more effectively on salient features, thereby enhancing detection performance and decreasing the false detection rate. In this study, the integration of the c2f-faster module and the MLCA attention mechanism allows the YOLOv8 model to more efficiently capture contextual information and detailed target features, thus enhancing the accuracy and robustness of detection. The enhanced structure of YOLOv8 is illustrated in **Figure 5**.

The specific execution process of the FastMLCA-YOLOv8 model is as follows:

1. Prior to being input into FastMLCA-YOLOv8, the image is resized to 640 × 640 × 3. The input image undergoes feature extraction by the backbone network to obtain a series of feature maps at different scales;
2. Subsequent feature learning and compression are executed using Fasternet’s bottleneck structure;
3. Incorporate the MLCA attention mechanism following the backbone network.;
4. Feature maps enhanced by the MLCA attention mechanism are sent to the detection head for target classification and bounding box regression;
5. At each scale, bounding boxes are filtered through the non-maximum suppression (NMS) algorithm to eliminate redundant detection results;
6. Ultimately, bounding boxes processed by NMS are rescaled to the original image dimensions, and the final target detection results are produced.

#### 2.3.3 FastMLCA-YOLOv8 model to recognize fruit stalks

**Figure 6:**
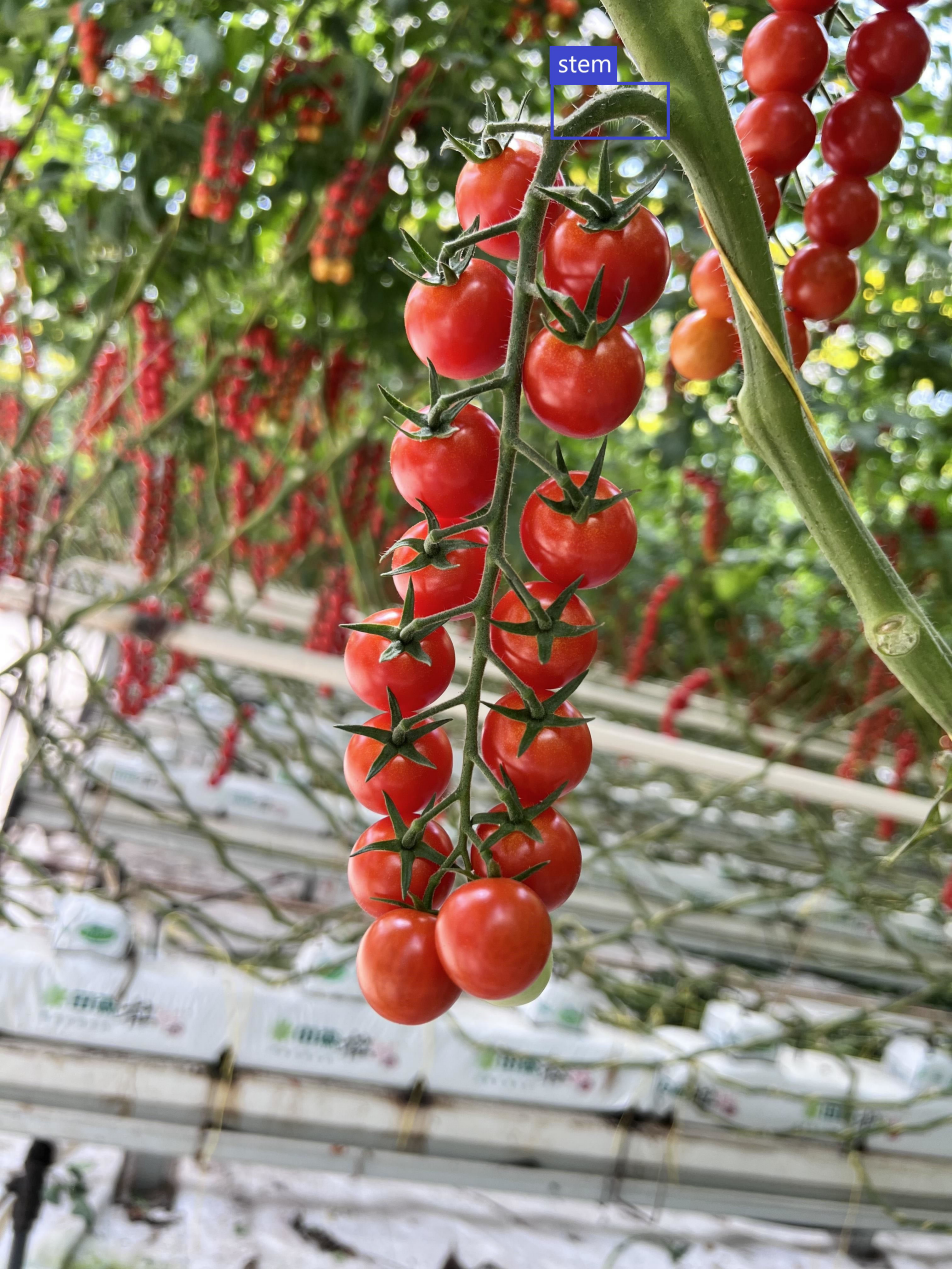
FastMLCA-YOLOv8 training result plot.

The FastMLCA-YOLOv8 model environment was established on Ubuntu for training and analysis purposes. The dataset annotations were converted from XML to TXT format, and the annotated dataset comprising 10180 entries was partitioned into training, validation, and test sets at a ratio of 8:1:1. The data utilized for the model comprised sample images varying in resolution, size, saturation, and angle. The model parameters were adjusted before running (Zhaoxin et al., 2022), with epchos modified to 200 and batch-size to 64, and the specific parameter settings are shown in **Table 2**. Under NVIDIA GeForce RTX 3090, during running, the model training time is only 3.203 hours. Of this, preprocessing takes 0.9 ms, inference takes 2.0 ms, loss calculation is 0.0 ms, and postprocessing per image takes 0.8 ms. The inference speed (FPS) reaches 270.3 fps, which can meet the demand for real-time detection of string tomato picking robots. The results obtained are demonstrated in **Figure 6**.

**Table 2:** Parameter description table.

Hyperparameterisation	Value	Clarification
Classes	stem	Category of identification
Image Size	640×640×3	Input Image Size
Epochs	200	Training Round
Batch-Size	64	Amount of data processed per batch
Workers	16	Controls the number of working threads of the data loader
Stride	1	Step Size Setting
Activation-Function	SiLU	Specific use of activation function types

#### 2.3.4 Extraction of fruiting peduncle ROI regions

**Figure 7:**
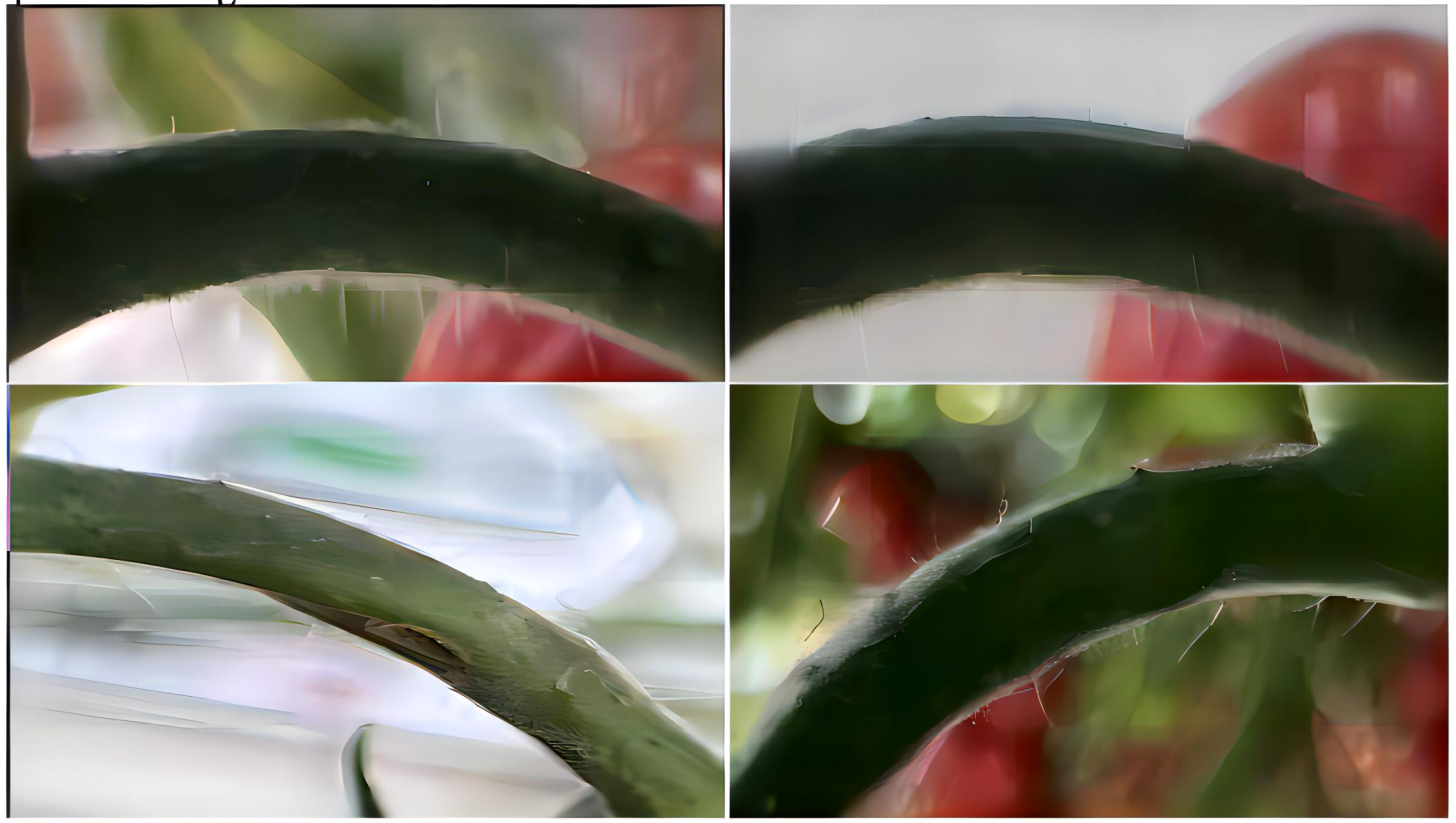
Fruit stalk ROI region extraction map.

Following the identification of fruit stalks in the tomato image via the FastMLCA-YOLOv8 algorithm, an extraction algorithm isolates the fruit stalk region as the Region of Interest (ROI) for further processing. The image, post fruit stalk extraction, is depicted in **Figure 7**.

#### 2.3.5 Refinement of the skeletal line of the fruiting peduncle

After using the ROI region extraction algorithm, the rectangular box where the pickable fruit stalks are located can be extracted, but it still can’t meet the demand of robotic picking, and it needs to be further researched on the fruit stalk region. In comparison to traditional segmentation algorithms like Otsu and Watershed, the convolutional neural network-based segmentation algorithm exhibits good robustness and can adapt to different lighting scenarios, thus this study adopts the improved K-means segmentation algorithm for segmenting the ROI region (Chakraborty et al., 2024).

The K-means algorithm uses distance as a criterion for assessing similarity. In other words, the shorter the distance between data objects, the more similar they are and the more likely they are to belong to the same category. The K-means algorithm operates as follows: in order to form the initial centres of the k groups, k data objects are first randomly selected from the dataset; the relative distance between each data object and the centre of the cluster in which it is located is then computed, and after that, the data objects are classified as being closest to the centre of the cluster; Eventually, the centre of each cluster is redefined by updating the centre of each cluster and adopting the average of all objects in the cluster as the new centre. The previous steps are repeated until the values of both the new and original cluster centres fall below a certain threshold, at which point the algorithm terminates.

**Figure 8:**
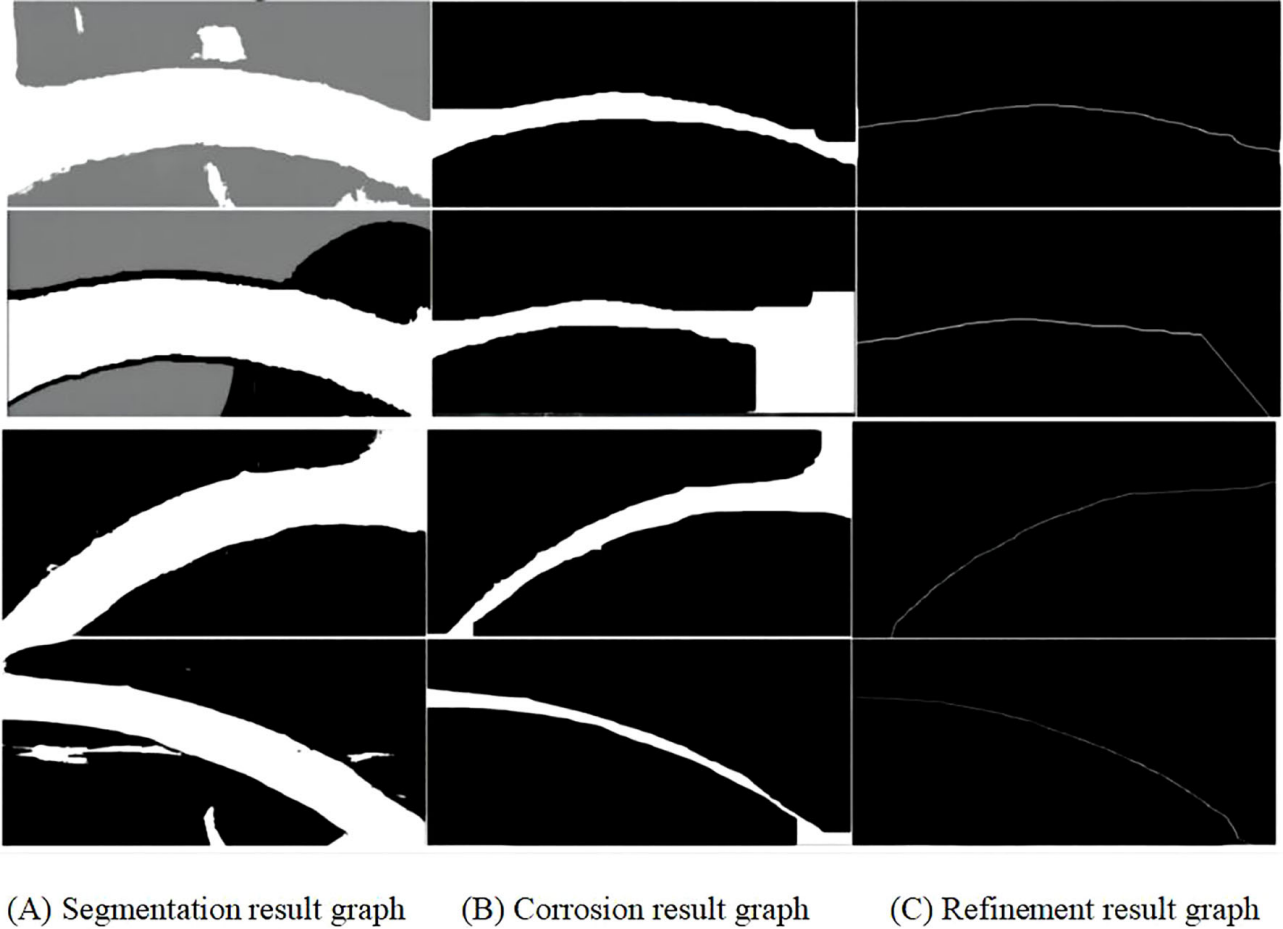
Resulting plot of segmentation, erosion and refinement.

In the improved K-means algorithm, K-means++ is used for cluster centre initialisation during cluster initialisation, where K-means++ randomly selects samples as the first cluster centre. Next, the sum of the squares of the shortest distances from all the remaining samples to the existing clustering centres is computed, and the next clustering centre is selected based on this probability distribution. Then, different numbers of clusters are tested in a loop, and Silhouette scores are calculated at each value of k, with the maximum average Silhouette score selected as the optimal number of clusters. The optimal number of clusters is determined by adding the silhouette coefficient to sil_scores + 2. Finally, the K-means object is reinitialized and fitted to the data to obtain the labels and reverts them to the shape of the image. Throughout the clustering process, by setting the parameter n_init to 10, the K-means algorithm will be iterated 10 times and the clustering result that minimises the SSE will be chosen as the output, the segmentation result is shown in **Figure 8A**.

After segmenting the fruit stalks using the improved K-means algorithm, there are still some isolated small spots in the image and noise problems such as burrs on the surface of the fruit stalks, internal holes, etc., which need to be processed using the corrosion operation to completely segment the background from the fruit stalks, and the corrosion results are shown in **Figure 8B**. The corrosion operation shrinks each subset B+A in the image A that corresponds exactly to the structural element B, as shown in Equation 2.


(2)  S=A⊗B={x,y|(B)xy⊆A}

The corroded fruit stalk images were then refined using the skeletonised zhang refinement algorithm. The zhang refinement algorithm is performed iteratively and all non-zero pixels need to be read each time the algorithm is run. In deciding whether to delete or retain each pixel (P1), close attention must be paid to the specific values of the eight pixels (P2 P3 P4 P5 P6 P7 P8) in its vicinity. Meanwhile, in the refinement process, the endpoint judgement condition is added, if only one pixel point in the 8-neighbourhood of a pixel point is a foreground pixel point except the point itself, i.e., the other 7 pixel points are background pixel points, then this pixel point can be considered as an endpoint, and the result of the refinement is shown in **Figure 8C**. By judging and deleting these endpoints, the main line strips can be better preserved and the fruit stalks can be refined completely.

#### 2.3.6 Obtaining picking point coordinate information

**Figure 9:**
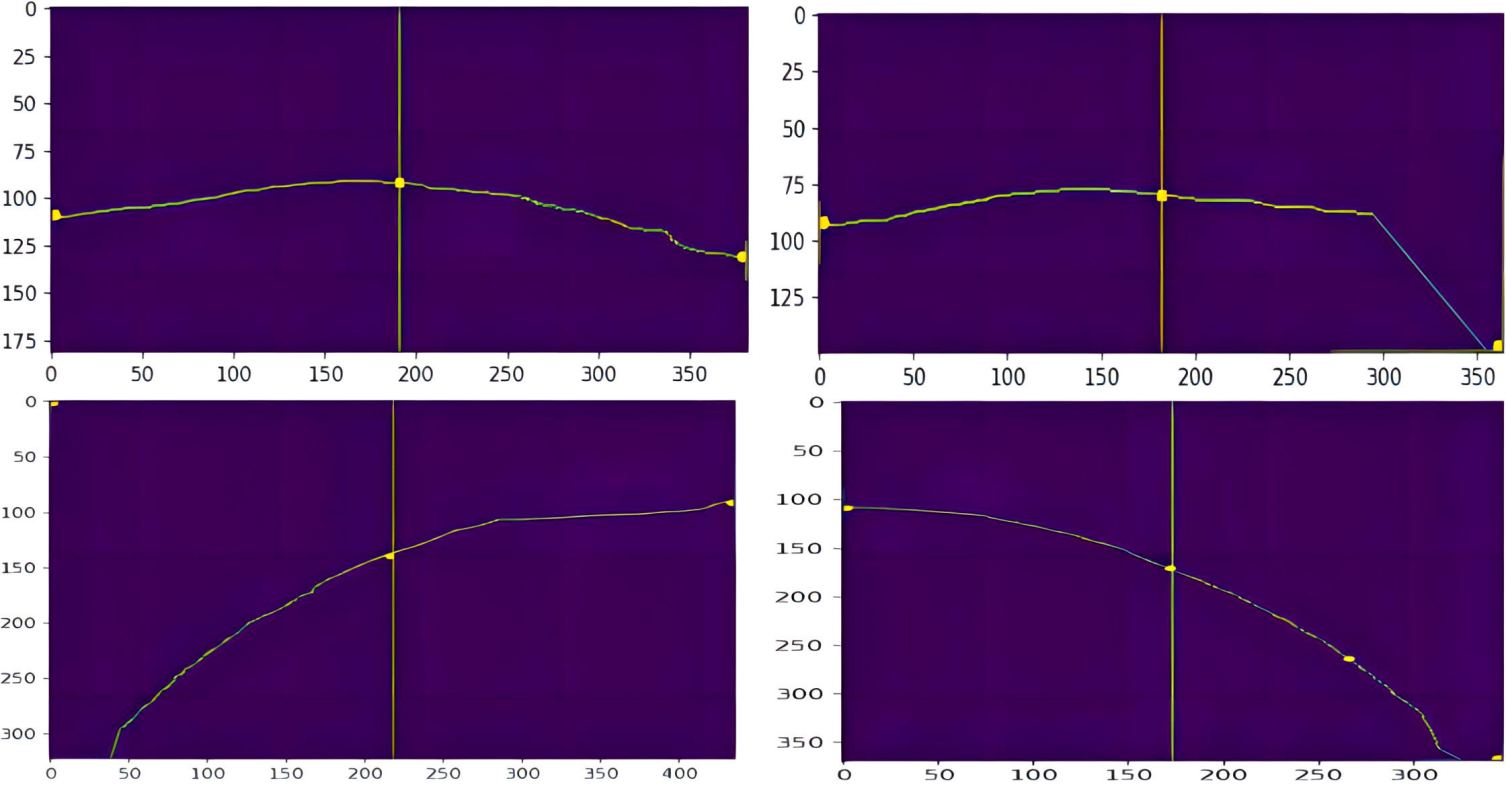
Picking point coordinate information map.

To prevent end-effector damage to the fruit stalks and main stem, short distances were prioritized during the picking process (Chen et al., 2023). The picking point was designated at the centre of the fruit stalk refinement map. In the skeletonized image, the clustered tomato fruit stalks extend almost from the top to the bottom. The precise location of the picking point is identified by the intersection of the image’s top and bottom centre lines with the fruit stalk’s skeletal line. The skeletonized image is converted to binary format, with pixel values of 1 assigned to the skeletal structures and 0 to others. By analysing pixel values along the centre line, the coordinates of the picking point in the skeletonized image are ascertained, as illustrated in **Figure 9**.

#### 2.3.7 Secondary extraction method to obtain depth values

After obtaining the 2D coordinate information of the fruit stalk picking point, the depth value and 3D coordinate information of the picking point (Bai et al., 2023) need to be further determined. Depth information for normally growing fruit stalks can be directly obtained using an RGB-D depth camera. However, in practical applications, fruit stalks may exhibit slender or overlapping characteristics, leading to issues with the depth camera failing to obtain depth information and duplicated identification of fruit stalks during depth acquisition. Consequently, this study introduces a secondary extraction method to address these challenges.

**Figure 10:**
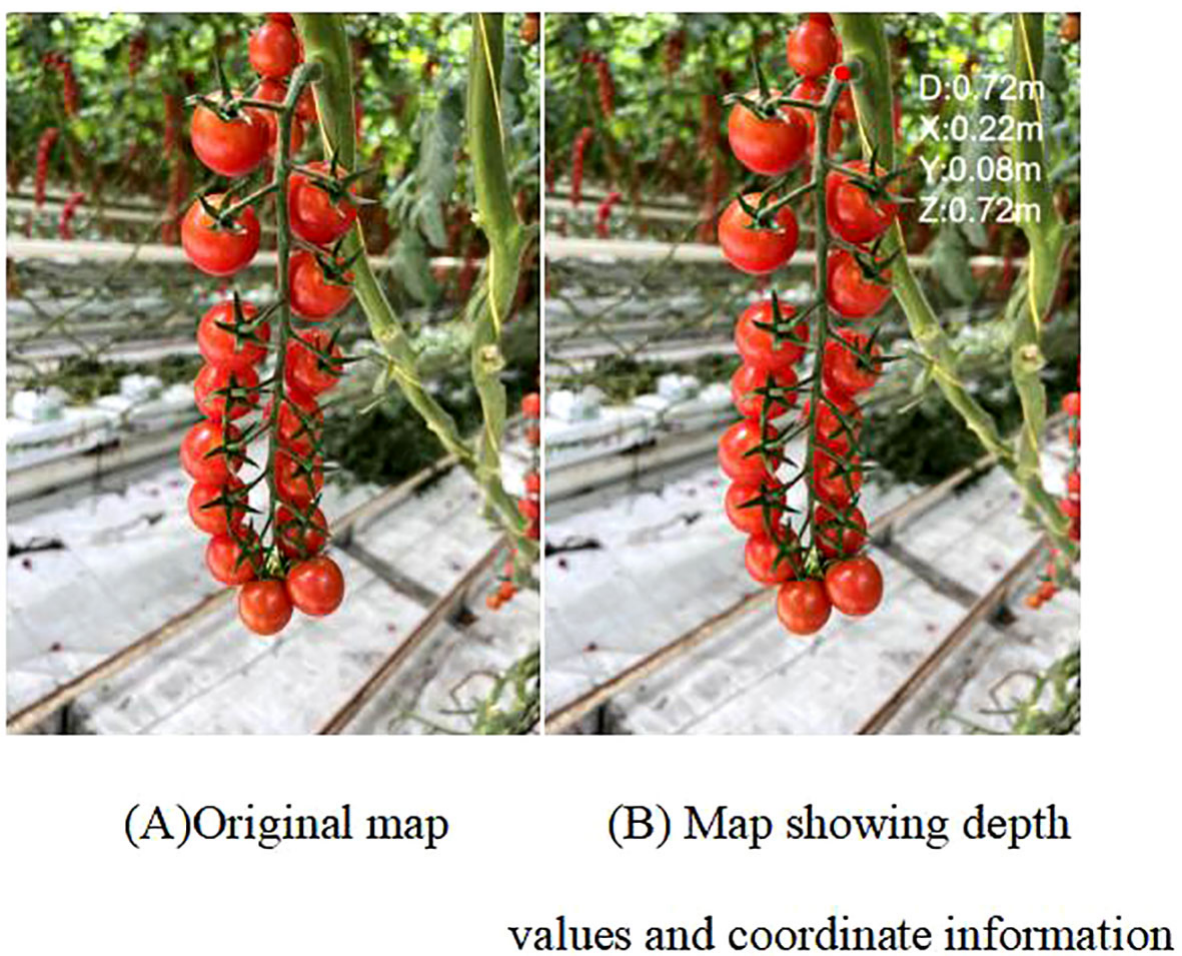
Picking points to obtain depth values. panel **(A)** Original map, **(B)** Map showing depth values and coordinate information.

The eroded fruit stalk image is converted into a binary map, where the white regions represent the fruit stalk with a value of 1, and the black background has a value of 0. The binarized image is subjected to dot multiplication with the fruit stalk depth image, facilitating the extraction of depth data from the fruit stalk region, labelled as {n0}. Considering the errors in depth values and the imprecision in fruit stalk region segmentation, the extracted depth set {n0} undergoes validity analysis, excluding depth ranges between 400-1000 mm to derive the residual depth set {n1}. The average value D1 of the depth set {n1} is calculated, designating the initial picking point depth value as D. The absolute difference between D1 and D is compared with the reference value k. If |D1-D|≤k, D is selected as the picking point’s depth value; if |D1-D|>k, D1 is selected instead. The reference value k can be experimentally determined as the maximum value of |D1-D|. Following the transformation and correction of the depth value acquired by the RGB-D depth camera, the 3D coordinate information (Ge et al., 2020) and depth value of the fruit stalk picking point are obtained, as depicted in **Figure 10**.

## 3 Results

### 3.1 FastMLCA-YOLOv8 result analysis

#### 3.1.1 Analysis of training results

**Figure 11:**
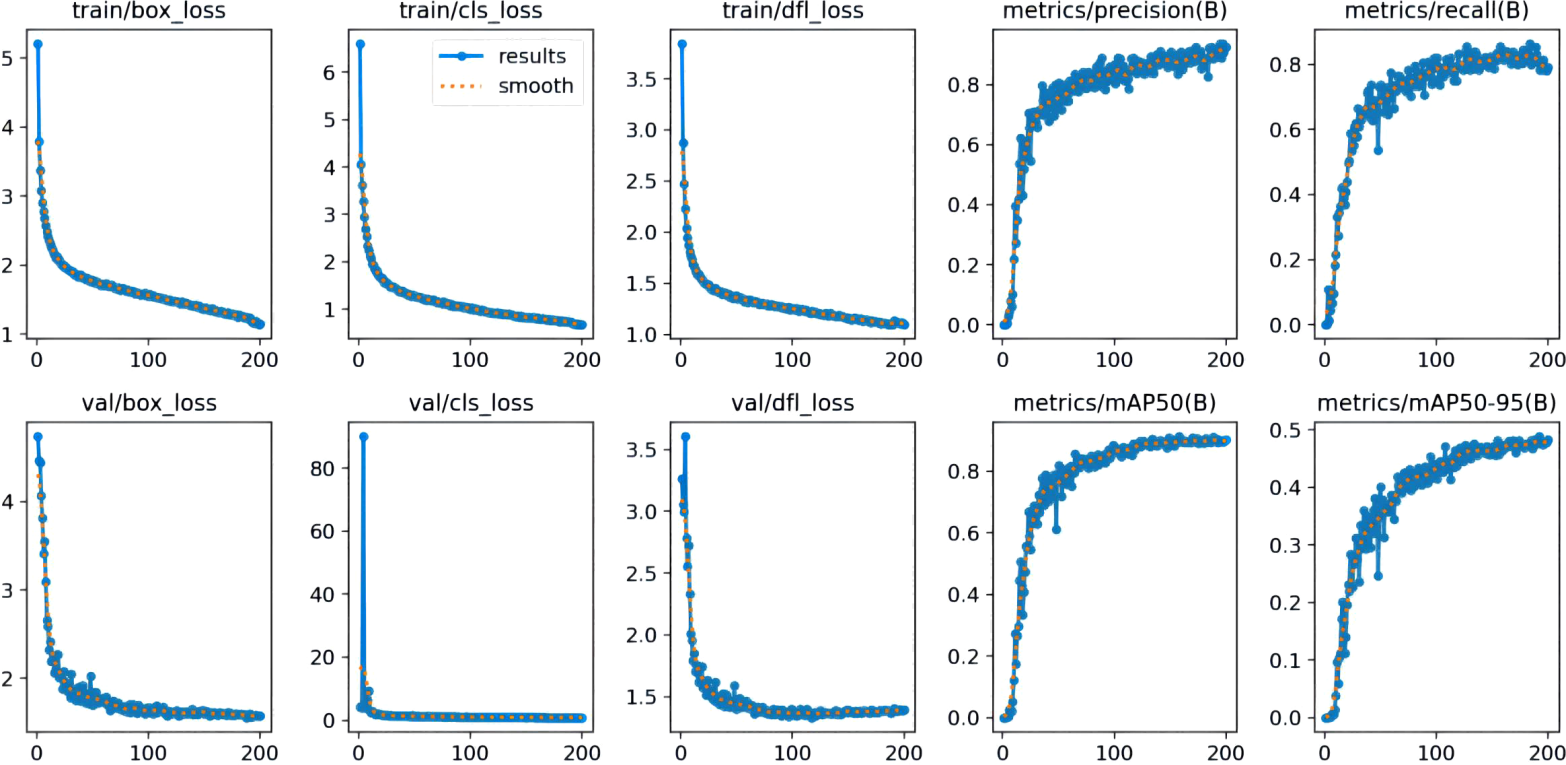
FastMLCA-YOLOv8 result.

The number of training rounds for the dataset using the FastMLCA-YOLOv8 model was set to 200 because the model iterates to 200 rounds to achieve the best results, stopping the training process. The RESULT results produced are shown in **Figure 11**.

Several key parameters for evaluating the model performance in the figure include bounding box loss, classification loss, feature point loss, precision, recall, and mean accuracy (mAP). The bounding box loss (box_loss) measures the positional accuracy of the predicted box by calculating the intersection and concurrency ratio (IOU) between the predicted box and the real box, which is converted into a loss value that reflects the accuracy of the model in locating the object. Classification loss (cls_loss), on the other hand, evaluates the classification performance of the model by comparing the difference between the predicted category distribution and the real category labels and then computing a loss value for classification. Feature point loss (dfl_loss) is employed to measure the disparity between predicted and actual feature points and evaluate the accuracy of feature point prediction. Furthermore, precision indicates the number of objects predicted by the model as positive examples that are actually real objects. This metric is utilized to measure the accuracy of the prediction results. Recall, on the other hand, refers to how many of all the objects that are actually positive cases are correctly detected by the model, reflecting the model's detection capability. mAP50 (mean accuracy at an IoU threshold of 0.5) evaluates the model's overall detection performance at lower IoU thresholds, while mAP50-95 (mean accuracy at IoU thresholds ranging from 0.5 to 0.95) provides model accuracies over a wider range of IoU thresholds, which are typically used to measure the overall performance of a model.

From the figure, it can be seen that the loss functions (Shuai et al., 2023) of both training and testing datasets are decreasing sharply, the loss function curve of val has stabilised at 100 rounds, and the loss function curves of train are all in a gradual process of decreasing. The curves of precision, recall,mAP50, and mAP50-95 are all gradually increasing, and converging to a steady state.

#### 3.1.2 YOLO model training results

Comparative analysis with various YOLO target detection models demonstrates that the FastMLCA-YOLOv8 algorithm excels in terms of recognition speed and accuracy. These comparative results are meticulously documented in **Table 3**.

**Table 3:** Comparison of YOLOv5, YOLOv6, YOLOv7, YOLOv8 and FastMLCA-YOLOv8 training data.

Recognition Model	Precision P%	Recall Rate R%	mAP@.5%	mAP@.5:.95%	GFLOPs
YOLOv5	0.861	0.764	0.828	0.380	15.8
YOLOv6	0.845	0.795	0.858	0.411	16.7
YOLOv7	0.837	0.807	0.864	0.409	103.2
YOLOv8	0.865	0.794	0.885	0.433	28.4
FastMLCA-YOLOv8	0.902	0.831	0.913	0.473	14.4

**Figure 12:**
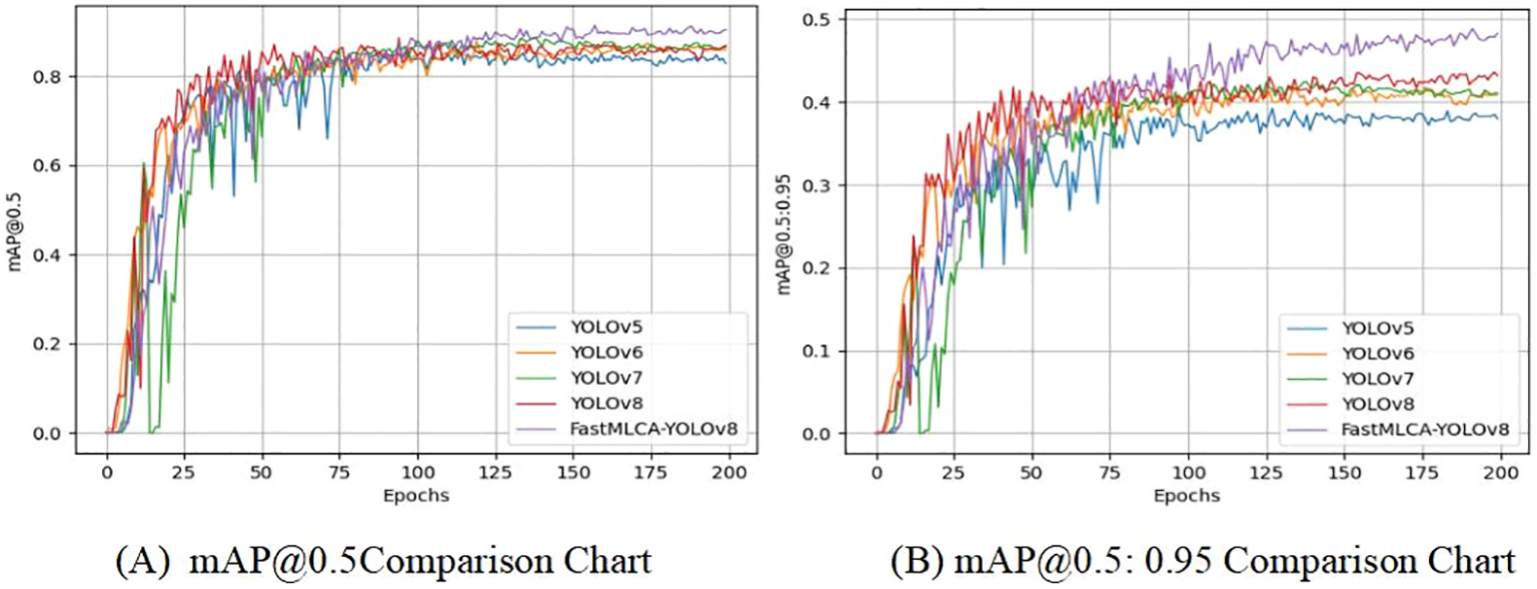
Comparison of training results. Panel **(A)** mAP@0.5comparison chart, **(B)** mAP@0.5:0.95comparison chart.

The table presents mAP@.5 scores for various YOLO target detection models: 0.828 for YOLOv5, 0.878 for YOLOv6, 0.864 for YOLOv7, and 0.885 for YOLOv8s. Remarkably, the FastMLCA-YOLOv8 model achieves an mAP@.5 score of 0.913, signifying a substantial 2.8% accuracy improvement compared to YOLOv8s. Then analyse the results obtained after running these models, as depicted in **Figure 12**.

Comparison of training results using detection model evaluation metrics that include the mAP@0.5 and mAP@0.5:0.95 metrics. The YOLOv5 curve experiences a relatively large decrease between rounds 25-75, before gradually stabilizing after round 75. Similarly, the YOLOv6 curve experiences a small decrease between rounds 75-125 and stabilizes after 125 rounds. In contrast, the YOLOv7 curve shows a substantial decrease up to round 25, with additional significant decreases between rounds 25-50, gradually converging to a relatively stable state after round 50. The YOLOv8s curve exhibits a substantial increase prior to 75 rounds, gradually stabilizing after 75 rounds, while the FastMLCA-YOLOv8 curve shows a gradual increase and stabilizes at 125 rounds (Zhu et al., 2023), reaching a relatively desirable level. In the mAP@0.5 graph, the mAP values for all models range between 0.8 and 1, yet the FastMLCA-YOLOv8s curve distinctly surpasses the other four. So the FastMLCA-YOLOv8 model recognised fruit stalks with higher accuracy and speed than other YOLO models.

**Figure 13:**
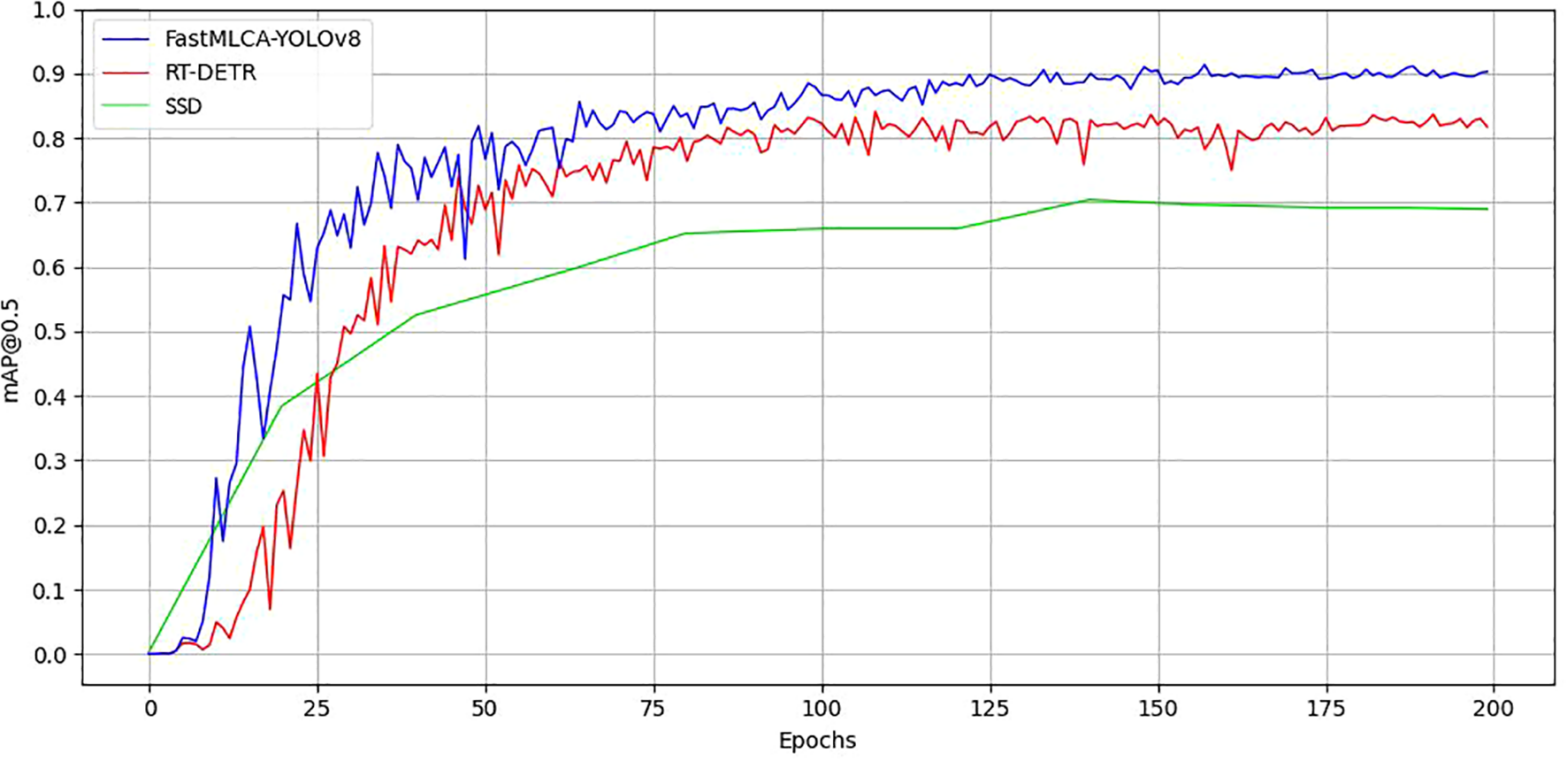
FastMLCA-YOLOv8 with SSD, RT-DETRmap@0.5 results chart.

To further validate the high accuracy and performance of the FastMLCA-YOLOv8 model, we conducted training sessions with the more advanced SSD algorithm and RT-DETR model on the same dataset for an equal number of epochs. The results are presented in **Table 4** and **Figure 13**. From the table, it can be observed that the SSD model, due to its characteristics of utilizing multi-scale feature maps and predicting multiple prior boxes, achieved a precision (P%) and mAP@.5:.95% of 0.922 and 0.611, respectively, which are 2 percentage points and 13.8 percentage points higher than those of the FastMLCA-YOLOv8 model. However, its recall rate (R%) and mAP@.5% were lower by 48.4 and 22.6 percentage points, respectively, compared to the FastMLCA-YOLOv8 model. Additionally, the SSD model’s computational resource consumption exceeded that of the FastMLCA-YOLOv8 model by 11.5 GFLOPs. Overall comparison reveals that the SSD model exhibits lower recall and precision rates compared to the FastMLCA-YOLOv8 model, and it consumes more computational resources. In comparison to the FastMLCA-YOLOv8 model, the RT-DETR model demonstrates lower precision (P%), recall (R%), mAP@.5%, and mAP@.5:.95% by 10.1, 7.1, 7.2, and 6.6 percentage points, respectively. Furthermore, the RT-DETR model consumes 86.2 more GFLOPs than the FastMLCA-YOLOv8 model. Overall, the RT-DETR model exhibits inferior performance in terms of precision, recall, and average precision, while requiring more computational resources. Therefore, the FastMLCA-YOLOv8 model demonstrates excellent recall and precision performance with lower computational resource consumption, enabling rapid and accurate identification of fruit stems, thus meeting the requirements for robotic detection tasks.

**Table 4:** Comparison of FastMLCA-YOLOv8 with SSD and RT-DETR Training Data.

Recognition Model	Precision P%	Recall Rate R%	mAP@.5%	mAP@.5:.95%	GFLOPs
FastMLCA-YOLOv8	0.902	0.831	0.913	0.473	14.4
SSD	0.922	0.343	0.687	0.611	25.9
RT-DETR	0.801	0.760	0.841	0.407	100.6

FastMLCA-YOLOv8 model performs better in terms of mAP@0.5 compared to YOLOv5, YOLOv6, YOLOv7, YOLOv8, and RT-DETR models, with improvements of 8.5, 3.5, 4.9, 2.8, and 22.6 percentage points, respectively. These results highlight the FastMLCA-YOLOv8 model’s superior recognition rate, specifically enabling accurate identification of fruit stalks. Additionally, its GFLOPs (Giga Floating Point Operations Per Second) value of 14.4 is the smallest among the compared models, proving that the FastMLCA-YOLOv8 model requires less computational resources to support its operation compared to other models. However, in the mAP@0.5:0.95% comparison, the FastMLCA-YOLOv8 model shows improvements of 9.3, 5.8, 6.4, 4, and 6.6 percentage points compared to YOLOv5, YOLOv6, YOLOv7, YOLOv8, and RT-DETR, but a decrease of 13.8 percentage points compared to SSD. This indicates certain limitations in the detection performance of the FastMLCA-YOLOv8 model at IoU thresholds. Further enhancements are required to improve the detection performance and submit detection accuracy.

#### 3.1.3 Indicators for model evaluation

The primary metrics used to evaluate the detection model are mAP and FPS. mAP represents the mean average precision, derived from the model’s precision and recall, while FPS indicates the inference speed. In this study, average precision is quantified by an area AP value, encapsulated within a Precision-Recall curve, and the F1 score is determined as delineated subsequently. Precision, or the precision rate, is defined as the ratio of correctly identified positive samples to all samples labeled as positive by the modelRecall, or the recall rate, signifies the proportion of actual positive samples that the model correctly identifies as positive. The accuracy and completeness are defined as Equations 3–5, respectively.


(3) $$\text{Accuracy rate}:\text{ Precision }=\frac{\text{TP}}{\text{TP}+\text{FP}}$$

(4) $$\text{Check all rate }\left(\text{recall}\right):\text{ Recall }=\frac{\text{TP}}{\text{TP}+\text{FN}}$$

(5) $$\text{Flscore}=2\frac{\text{Precision}\times \text{Recall}}{\text{Precision}+\text{Recall}}$$

When the target is classified as positive and others are classified as negative, True Positives (TP) are instances where the target is correctly predicted as positive. False Negatives (FN) occur when a positive target is incorrectly predicted as negative. False Positives (FP) are instances where a negative target is incorrectly predicted as positive. True Negatives (TN) occur when a negative target is correctly predicted as negative.

### 3.2 Analysis of fruit stalk segmentation results

**Figure 14:**
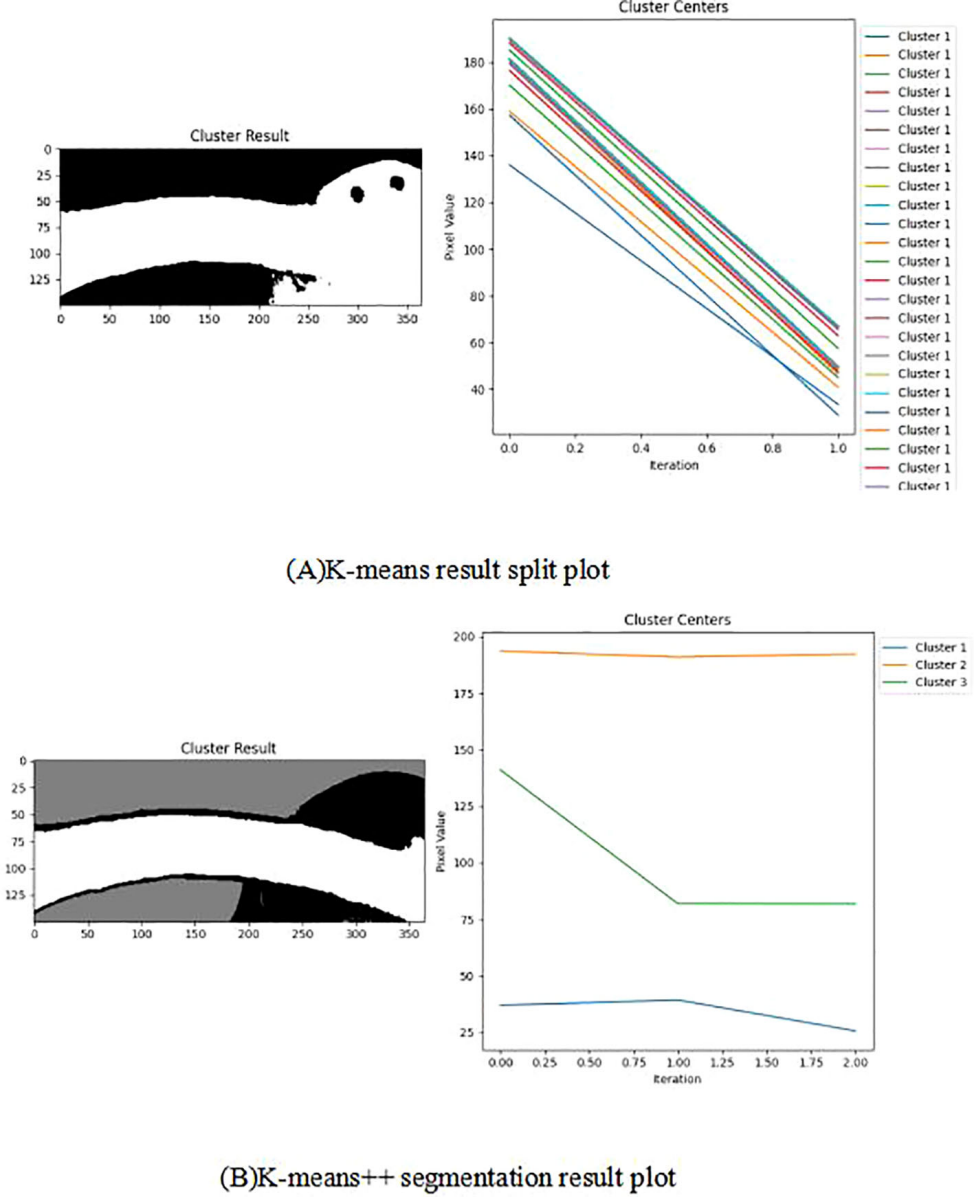
Comparison of improved K-means processing results with original K-means processing results. Panel **(A)** K-means result split plot, **(B)** K-means++ segmentation result plot.

The application of the K-means algorithm in segmenting fruit stalk images frequently introduces noise into the segmentation outcomes, prompting the adoption of an optimized and enhanced K-means algorithm for image segmentation. The enhanced K-means algorithm more precisely determines the initial clustering centre, mitigating the risk of converging to local optima and thus improving the clustering accuracy. The Silhouette coefficient autonomously identifies the optimal number of clusters, circumventing the subjectivity inherent in manual selection and ensuring greater clustering precision. **Figure 14** illustrates the comparative graph between the results processed by the improved and original K-means algorithms. The Cluster Result demonstrates that the improved K-means algorithm effectively differentiates the background from the fruit stalks, as well as the fruit stalks from the fruits. It is observable that while the original K-means algorithm requires multiple iterations to finalize clustering and segmentation, the improved K-means algorithm achieves clustering results in just three iterations. Furthermore, after one iteration, the pixel values of the cluster centre stabilize throughout the iterative process, resulting in improved clustering outcomes.

**Figure 15:**
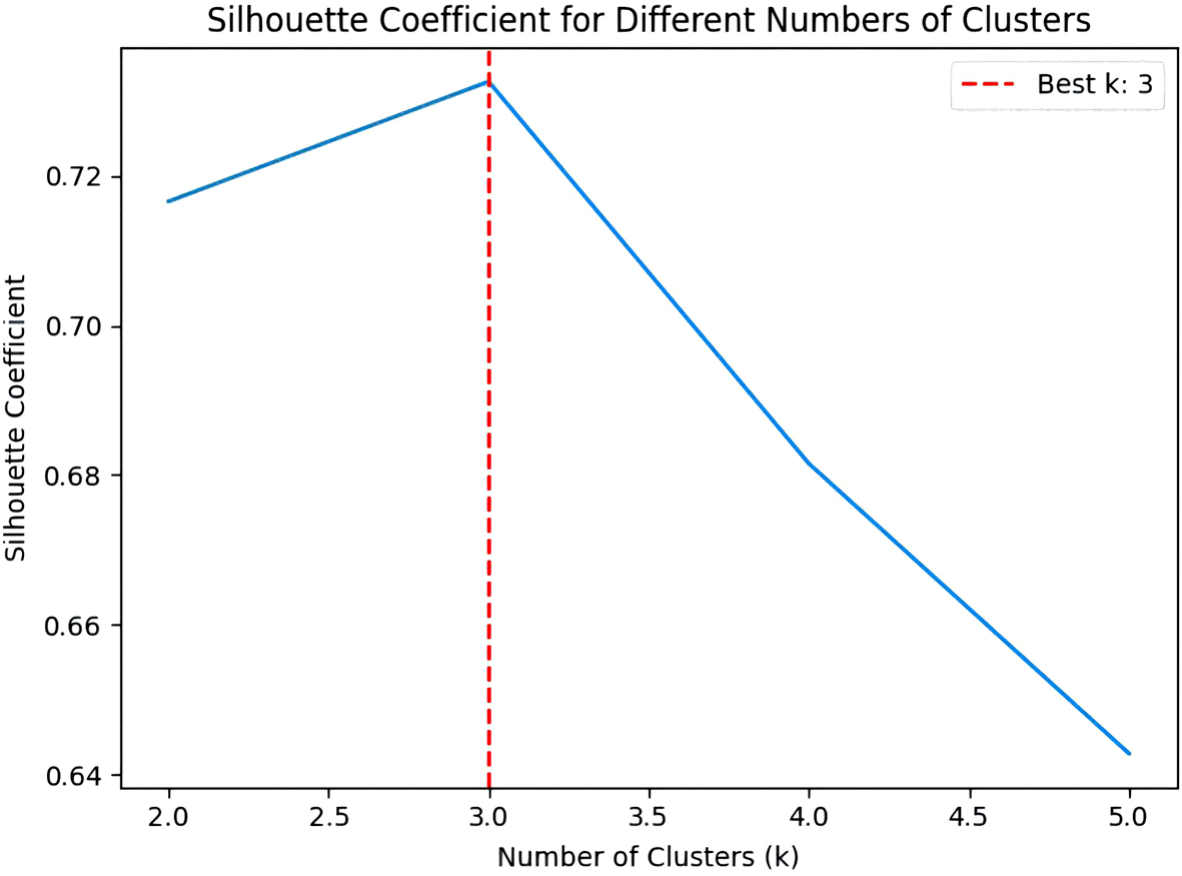
Silhouette coefficient processing results chart.

The line graph of the Silhouette coefficient elucidates the variation in the Silhouette coefficient across different cluster counts, with the x-axis representing the number of clusters (k) and the y-axis representing the Silhouette coefficient. In **Figure 15**, the red dotted line signifies that the optimal cluster count is 3, denoting that the Silhouette coefficient attains its maximum value at this cluster count.

### 3.3 Analysis of results for reference value k

To address the issue of missing depth information in fruit stalks, this study implements a secondary extraction method. Within this method, the reference value k was empirically determined using 10 randomly selected fruit stalks that possessed complete depth data.

The depth value derived from the secondary extraction method is denoted as D1, whereas the initial depth value obtained directly for the picking point is designated as D. Subsequently, the absolute difference between D1 and D is calculated, with the experimental findings summarized in **Table 5**.

**Table 5:** Comparison of depth values D1 obtained by secondary extraction method and original depth values D.

Serial number	Original Depth D/mm	Mean Depth D1/mm	Absolute Difference |D1-D|/mm
1	526.32	306.73	219.59
2	472.83	298.36	174.47
3	589.73	341.84	247.89
4	610.52	405.79	204.73
5	565.33	364.91	200.42
6	723.46	477.90	245.56
7	534.65	321.50	213.15
8	582.76	351.02	231.74
9	631.78	425.69	206.09
10	574.39	361.25	213.14

Data in **Table 5** indicate that the maximum value of |D1-D| is 247.89 mm, the minimum value is 174.47 mm, and the mean value is 215.68 mm. Consequently, k can be set to the mean value of 215.68 mm as the reference value. This indicates that the secondary extraction method not only effectively addresses the issue of missing depth values for fruit stalks in the depth map but also discerns the loss of depth information, thereby enhancing the precision of the depth value at the picking point.

### 3.4 Depth value error analysis

The distance between the fruit stalk and the end-effector’s shear centre point during the shearing process served as an evaluation criterion for assessing the positioning accuracy of the string tomato picking robot (Luo et al., 2018) at the picking point. The depth value of the picking point derived from the image was compared with the actual depth value, revealing an error range of ±2.5 mm. The corresponding error equation is presented in Equation 6. An analysis of the depth error at the picking point is detailed in **Table 6**.

**Table 6:** Depth value error analysis.

PanelActual Depth Value x*/mm	PanelCaptured Depth Value x/mm	PanelDepth Value Error ϵ(x) /mm
740.57	741.63	1.06
575.05	577.14	2.09
643.89	641.86	-2.03
662.32	664.21	1.89
710.59	708.65	-1.94
601.31	601.32	0.01
564.44	566.92	2.48
524.09	521.63	-2.46


(6) $$\text{ϵ}\left(\text{x}\right)=\text{x}-{\text{x}}^{*}$$

where ϵ(x) represents the absolute error, x represents the depth value acquired by the depth camera, and x* represents the actual depth value.

## 4 Discussion

The growth patterns of tomato fruit stalks in diverse environments result in varied growth attitudes, necessitating considerations of these attitudes, potential obstructions, or insufficient stalk length during the identification of picking points. Such factors can lead to unrecognizable fruit stalks or issues with missing depth values, requiring the formulation of appropriate solutions. This paper delves into the identification and localization of fruit stalks in hanging tomato bunches cultivated in greenhouse settings, providing an in-depth discussion and analysis.

Exploratory experiments aimed at enhancing the recognition rate of the YOLOv8model were conducted. A comparative analysis was performed on the training of the fruit stalk dataset using various YOLO models, leading to the adoption of an improved YOLOv8s target detection model for fruit stalk recognition. In a study to improve the YOLOv8 model and increase recognition rates. Tianyong Wu introduced the lightweight SEConv convolution in place of the standard convolution in the YOLOv8 model, reducing the network’s parameters, accelerating the detection process, and enhancing the algorithm’s performance (Wu and Dong, 2023). Shichu Li proposed the YOLOv8 - AFPN - M - C2f algorithm, which replaces the YOLOv8’s head with the AFPN - M - C2f network, enhancing the model’s sensitivity to smaller objects (Li et al., 2023) Yang G proposed an improved YOLOv8s-based automatic tomato detection method, replacing standard convolution with depth separable convolution (DSConv) and incorporating a DPAG module to enhance detection accuracy in complex environments (Yang et al., 2023). In this study, the YOLOv8 model is enhanced by replacing the Fasternet bottleneck with the C2f bottleneck and integrating the MLCA attention mechanism post-backbone network, thereby developing the FastMLCA-YOLOv8 model. This novel model excels in identifying fruit stalks that closely resemble main stems and leaves within complex scenes. Comparative analysis with other YOLO models, as illustrated in **Table 3**, reveals that the FastMLCA-YOLOv8 model achieves a recognition rate of 91.1%, successfully balancing speed and accuracy. Nonetheless, the improved model has certain limitations, such as its applicability in specific environments and dataset selection, which require further validation and refinement.

In order to improve the image segmentation accuracy, YanPing Zhao introduced a similarity calculation method addressing the K-means algorithm’s limitations, utilizing weighted and Euclidean distances. Experimental results demonstrate that this new algorithm surpasses the traditional K-means in efficiency, accuracy, and stability (Zhao and Zhou, 2021). Shyr-Shen Yu proposed a hierarchical approach with three-level and two-level K-means algorithms, where a robust set of initial clustering centres mitigates anomalies, enhancing data clustering accuracy (Yu et al., 2018). Chaturvedi E N introduced a novel K-means clustering algorithm that systematically calculates the initial centre of mass, improving both accuracy and processing time (Chaturvedi and Rajavat, 2013). To ensure successful separation of fruit stalks from the fruit stalk image, the K-means++ clustering method is applied to initialize cluster centres. Furthermore, Silhouette coefficients are used to automatically determine the optimal number of clusters for segmenting the fruit stalk region. The results demonstrate that the improved K-means segmentation algorithm not only effectively distinguishes the background from the fruit stalks but also accurately separates the fruit stalks from overlapping fruits, thus enhancing depth accuracy. However, this approach may face challenges when dealing with high-complexity scenes and may exhibit reduced segmentation performance under extreme lighting conditions. These limitations warrant further investigation and resolution in future research.

To solve the problem of missing depth values due to the limited accuracy of depth cameras. The quadratic extraction method in this paper is analysed in comparison with the algorithms proposed by other researchers. Satapathy Sukla addressed missing data in depth maps by employing hyperpixel division on the corresponding RGB image; the method estimates missing information in degraded observations through self-similarity across non-local patches within the hyperpixel search window (Satapathy and Sahay, 2021). Hsu H applied a supervised learning approach to address depth value discrepancies in colour images, effectively predicting depth values within gaps (Hsu et al., 2022). Ali M A used deep metric learning to make Mis GAN for multi-task missing data filling. The semantic representation of an image is extracted using an image feature extraction network and deep metric learning is performed to learn good feature embeddings by maximizing inter-class differences and minimizing intra-class differences. The proposed method is demonstrated to significantly outperform other methods by conducting several experiments on the dataset (Al-taezi et al., 2024). In this study, the secondary extraction method is employed to retrieve effective depth values from binarized images of fruit stalks and compare them with the original depth values of picking points to ascertain accurate depth measurements. This method achieves optimal depth determination for picking points, ensuring precision in depth estimation crucial for tomato harvesting applications. The findings underscore the method’s efficacy in tackling challenges related to the slender nature of fruit stalks and maintaining depth map integrity, thereby enhancing overall accuracy in depth estimation.

## 5 Conclusions

In this study, we chose the fruit stalks of bunch tomatoes grown by hanging in greenhouses as the research object.Specifically, we conducted an in-depth investigation into the visual localisation of the picking position for bunch tomatoes. To address the challenge of identifying and locating the picking points of bunch tomatoes in complex environments, a picking point identification and localisation method based on FastMLCA-YOLOv8 and RGB-D information fusion is proposed, which initially constructs a FastMLCA-YOLOv8 model for identifying the fruit stalks of bunch tomatoes; subsequently, a cropping algorithm is used to crop the fruit stalks individually out of the bunch tomato image, and then a improved K-means, corrosion algorithm, zhang refinement and other algorithms are used to segment, denoise and refine the skeletal lines of the fruit stalk region to obtain the specific coordinate information of the picking point of the string tomato in the image; finally, the depth value of the fruit stalk is extracted by using the RGB-D depth camera and the secondary extraction method to obtain the three-dimensional coordinate information and depth value of the picking point. The results show that this study achieves 91.1% recognition rate for fruit stalks, which improves the accuracy by 2.8% compared to the YOLv8 model. The improved K-means algorithm is able to completely separate the fruit stalk region from the background region compared to the original algorithm. The error range of the depth value is only ±2.5 mm, which provides the necessary data support for the picking robot. Simultaneously, the identification and localisation method proposed in this study is not only applicable to the picking points of tomato bunches, but also applicable to the identification and localisation of picking points of other bunches of harvested fruits in complex environments. Nevertheless, there are some limitations in this study,future efforts will focus on improving and optimizing the picking point identification and localization method to enhance system performance and stability.

In this study, ripe bunch tomato fruit stalks grown by hanging in greenhouse greenhouses were selected as the research object, and the picking location of bunch tomatoes was studied in depth for visual localisation. In order to solve the problem of identifying and locating the picking points of string tomatoes in complex environments, an improved YOLOv8 and depth camera fusion method for identifying and locating the picking points of string tomato fruit stalks is proposed, which firstly constructs a FastMLCA-YOLOv8 model for identifying the fruit stalks of ripe string tomatoes; after that, a cropping algorithm is used to crop the fruit stalks individually out of the string tomato images, and then an improved After that, the fruit stalk is cropped out from the bunch tomato image using the cropping algorithm; then the fruit stalk is segmented, denoised and the skeletal lines are refined using the improved K-means, corrosion algorithm and zhang refinement algorithms to obtain the specific coordinate information of the picking point of the bunch tomato in the image; finally, the depth value of the fruit stalk is extracted using the RGB-D depth camera and the quadratic extraction method, and then the three-dimensional coordinate information and the depth value of the picking point are obtained. The results indicate that the recognition rate of fruit stalks in this study reaches 91.1%, which represents a 2.8% improvement in accuracy compared to the YOLOv8 model. The improved K-means algorithm can completely separate the fruit stalk region from the background region compared to the original algorithm. The depth value error is limited to ±2.5 mm, providing essential data support for the picking robot. Simultaneously, the identification and localization method proposed in this study is not only applicable to the identification and localization of the picking points of string tomatoes but also to the identification and localization of the picking points of other string-harvested fruits in complex environments.

However, there are some limitations in this study, and subsequently we will further enhance the accuracy and stability of detecting targets at fruit stalk picking points of bunch tomatoes to ensure that the targets can be effectively identified and localized in a variety of complex scenarios. Secondly, the current detection method for fruit stalk picking points has challenges in dealing with the situation where the fruit stalks are occluded, and further research and improvement of the algorithm are 646 needed. In the future, we will focus our research on the study of occluded fruit stalks and fruit stalks that are too short to accurately find the picking point, and reduce the error of the depth camera in acquiring the depth value. Finally, the proposed method will be refined and optimized to enhance the identification and localization of picking points for various fruits and vegetables.

## Data Availability

The raw data supporting the conclusions of this article will be made available by the authors, without undue reservation.
